# Success of anti-CD20 monoclonal antibody treatment for severe autoimmune hemolytic anemia caused by warm-reactive immunoglobulin A, immunoglobulin G, and immunoglobulin M autoantibodies in a child: a case report

**DOI:** 10.1186/s13256-017-1449-2

**Published:** 2017-11-14

**Authors:** Houda Ajmi, Sameh Mabrouk, Saida Hassayoun, Haifa Regaieg, Minyar Tfifha, Chemli Jalel, Hadef Skouri, Noura Zouari, Saoussan Abroug

**Affiliations:** 1 Pediatrics Department, Sahloul Teaching Hospital, 4054 Sousse, Tunisia; 2 Hematology Department, Farhat Hached Teaching Hospital, 4031 Sousse, Tunisia; 3 Hematology Laboratory, Sahloul Teaching Hospital, 4054 Sousse, Tunisia

**Keywords:** Autoimmune hemolytic anemia, Steroid-resistant, Rituximab, Warm IgM autoantibody

## Abstract

**Background:**

Autoimmune hemolytic anemia is rare in children. First-line therapies for this disease consist of corticosteroids and intravenously administered immunoglobulin that are effective in most patients. However, a small proportion of cases (5 to 10%) is refractory to these therapies and may represent a medical emergency, especially when hemolysis is due to warm immunoglobulin M. Recently, reports of the use of rituximab in adult autoimmune diseases have shown promising results. In children, there are few studies on the use of rituximab in the treatment for autoimmune hemolytic anemia, especially on its long-term efficacy and adverse effects.

**Case presentation:**

Here, we report the case of a 10-year-old Tunisian girl with refractory acute autoimmune hemolytic anemia caused by warm-reactive immunoglobulin A, immunoglobulin G, immunoglobulin M, and C3d autoantibodies. First-line treatments using corticosteroids and intravenously administered immunoglobulin were ineffective in controlling her severe disease. On the other hand, she was successfully treated with rituximab. In fact, her hemolytic anemia improved rapidly and no adverse effects were observed.

**Conclusions:**

The case that we report in this paper shows that rituximab could be an alternative therapeutic option in severe acute autoimmune hemolytic anemia with profound hemolysis refractory to conventional treatment. Moreover, it may preclude the use of plasmapheresis in such an urgent situation with a sustained remission.

## Background

Autoimmune hemolytic anemia (AIHA) is a rare acquired disorder with an estimated incidence of 1 to 3 per 100,000/year [1]. It is caused by autoantibodies, which lead to the destruction of the red blood cells (RBCs). Confirmation of this disease is based on the direct antiglobulin test (DAT) which detects the autoantibodies and/or complement on the surface of the RBCs [2]. Conventional therapies of AIHA include corticosteroids as a first-line therapy with a successful response in 70 to 85% of cases [3]. Some patients remain refractory or corticosteroid dependent and require second-line therapies such as immunosuppressive drugs and splenectomy. However, these treatments are not always effective, especially in an urgent situation, and convey a great risk of infection [4].

We report in this paper a case of acute life-threatening AIHA in a 10-year-old child. The disease was refractory to corticosteroids and immunoglobulin treatment, and responded successfully to rituximab.

## Case presentation

A 10-year-old Tunisian girl presented to our department with fever, vomiting, and asthenia. She had neither family history of hematological diseases nor other illness before the disease onset. During her admission, a physical examination showed a child of normal stature with pallor and fever (body temperature of 39 °C). She was tachycardic at 141 beats/minute and her blood pressure was at 107/51 mmHg. She had tachypnea at 52 breaths/minute with oxygen saturation in air at 95% and no heart murmur. An abdominal examination found an isolated splenomegaly. Biochemical analysis showed signs of hemolysis: total bilirubin, 51 microml/L with a direct fraction of 9 microml/L; haptoglobin < 58 mg/L; and D-lactate dehydrogenase, 2051 U/L. Her complete blood count showed severe anemia with hemoglobin (Hb), 2.6 g/dl; RBCs, 90 × 10^4^/mm^3^; hematocrit (Ht), 6.6%; mean corpuscular volume (MCV), 91.3 fL; mean corpuscular hemoglobin (MCH), 31 pg; mean corpuscular hemoglobin concentration (MCHC), 33 g/dL; reticulocytosis, 239,400/mm^3^; white blood cells, 40,500/mm^3^; and platelets, 540,000/mm^3^. The blood smear did not reveal schistocytes. A DAT identified autoantibodies of the immunoglobulin M (IgM), immunoglobulin A (IgA), immunoglobulin G (IgG) isotypes, and C3d that reacted strongly (3+) at 37 °C with all tested cells. This confirmed the diagnosis of AIHA.

**Fig. 1:**
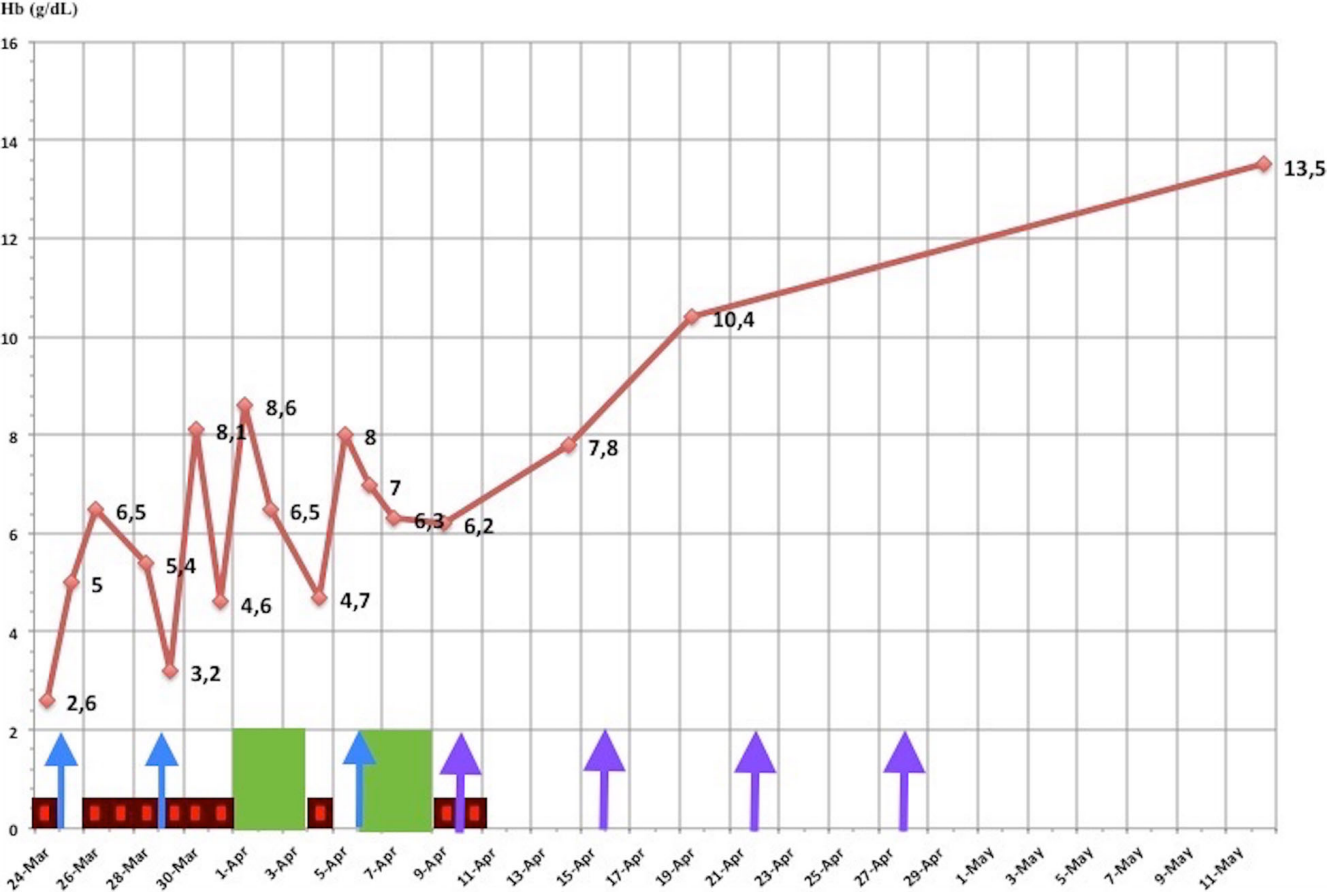
Effect of different therapies used in our patient on hemoglobin levels. *Hb* hemoglobin (g/dL), intravenously administered immunoglobulin (1 treatment is 1 g/kg per day for 2 days),  methylprednisolone (1 treatment is 30 mg/kg per day for 3 days), RBCs  red blood cells transfusion, and  rituximab (1 dose is 375 mg/m^2^)

Bacteriological analysis performed to access the etiology of the fever showed urinary infection (the urine analysis showed leukocytes of 2000/mm^3^ and erythrocytes of 1000/mm^3^) with C-reactive protein at 206 mg/L. The urine culture was negative because the child had already received amoxicillin. Then, antibiotherapy was continued with intravenously administered cefotaxime and amikacin. For AIHA, she had intravenously administered immunoglobulin (IVIG) at a dose of 1 g/kg per day twice during 48 hours. She also received repetitive RBCs transfusion. After controlling the infectious process, she was given a high-dose pulse of methylprednisolone (30 mg/kg per day for 3 days) followed by orally administered prednisone at a dose of 2 mg/kg per day. Due to the persistence of altered neurological status and severe hemolysis, she was again given IVIG and high-dose pulse methylprednisolone but without any benefit. Therefore, it was urgent to use a second-line therapy. In view of severe life-threatening hemolysis and her age, rituximab was deemed a better option than plasmapheresis, splenectomy, and/or cytotoxic drugs. Rituximab was given at a dose of 375 mg/m^2^ per week for 4 weeks. Afterward, prednisone was continued. After the second dose of rituximab our patient no longer required blood transfusions and there was a slow rise in her Hb level until day 15 at which point it stabilized (Fig. 1). At the last out-patient follow up (4 months after her hospitalization), her Hb and hemolytic markers were still within a normal range: Hb, 13 g/dl; RBCs, 4.31 × 10^6^/mm^3^; Ht, 37.5%; and reticulocytosis of 69,000/mm^3^. Biological investigations (serological tests, antinuclear antibodies screening, and immunity exploration) done to search for an underlying condition such as infection, lupus, or immune deficiency disease were all negative.

## Discussion

Warm AIHA is usually caused by IgG antibodies. Cases of AIHA caused by other autoantibodies, such as warm-reactive IgM or IgA, have rarely been reported in the literature [5]. In these patients, often, warm IgM autoantibodies and warm IgA autoantibodies are found either alone or in combination with IgG autoantibodies [5]. AIHA caused by both warm-reactive IgM and IgA autoantibodies is exceedingly rare [5]. Branstetter *et al*. [5] reported the first case of severe AIHA caused by warm-reactive IgM and IgA autoantibodies in an otherwise healthy 3-month-old child. In our case, we highlight an unusual combination of autoantibodies causing AIHA in a pediatric patient. In fact, the DAT showed the presence of multiple warm-reactive autoantibodies composed of IgM, IgG, IgA, and C3d autoantibodies. The presence of warm IgM antibodies could explain the severity of our case and especially the resistance to steroids [5–7]. In fact, unlike typical warm-reactive IgG-mediated AIHA, warm-reactive IgM-mediated AIHA is often steroid refractory and unresponsive to IVIG [5]. Splenectomy and rituximab are the only second-line treatments that could be used in these cases with a proven short-term efficacy [4]. However, splenectomy is associated with surgical and infective complications. Rituximab is a humanized chimeric anti-CD20 monoclonal antibody, directed against CD20, which rapidly depletes B cells from the blood, lymph nodes, and bone marrow [8]. Its *in vivo* mechanism of action includes complement-mediated cytotoxicity, antibody-dependent cytotoxicity, apoptosis, and inhibition of B cell proliferation [8, 9]. The dosage used is 375 mg/m^2^ administered weekly for 4 weeks in total. This drug was first developed for the treatment of hematological malignancies, such as aggressive non-Hodgkin lymphoma and chronic lymphocytic leukemia. The efficacy of rituximab in the treatment of pediatric patients with AIHA has been proved in a few studies [9–11]. The largest series of AIHA treated by rituximab in children was described by Zecca *et al*. [10]. In this study 15 children with refractory AIHA were treated with rituximab. Of these 15 children, 13 (87%) responded, whereas 2 patients did not show any improvement. Two months after treatment, the median Hb level of patients had increased from 7.7 g/dL to 11.8 g/dL and the median absolute reticulocyte count had decreased from 236 to 109 × 10^9^/L. The interval from diagnosis to treatment ranged from 2.1 to 98.5 months and the interval from treatment to response varied from 5 to 72 days. All children received two or more courses of immunosuppressive treatment, and a splenectomy was performed on two patients in order to control the hemolysis. Only three responder patients relapsed and received a second course of rituximab, achieving a disease remission. Quartier *et al*. [9] evaluated the efficacy of rituximab in a group of six children with AIHA. The duration of AIHA before the use of rituximab varied from 3 to 10 months. All patients achieved sustained remission after rituximab. In these two studies, patients had no severe hemolysis that could threaten their life. The type of anemia was warm-reactive IgG-mediated AIHA in the majority of cases, except one of them who had cold-reactive IgM-mediated AIHA. Our observation is particular because her anemia was very severe and poorly tolerated. The severity of the hemolysis in this case could be explained by the presence of warm IgM autoantibodies. The child required immediate treatment with multiple packed RBCs transfusions (at least one transfusion/day), IVIG, and corticosteroids. However, all of these treatments failed. Plasmapheresis may be considered in such severe forms. However, it has a short-term profit, a high rate of morbidity, and mortality. Thus, we decided to treat our patient with rituximab. This drug is effective in acute AIHA with profound hemolysis refractory to conventional therapies. Moreover, it may spare the patient from plasmapheresis in such a critical situation with a sustained remission in most cases.

## Conclusions

Refractory acute AIHA is a severe disease. Its management is difficult and require the use of high doses of corticosteroid, immunosupressive therapy, plasmapheresis or splenectomy. Actually rituximab is an effective treatment for this disease that induce remission in most pediatric patients with a sustained response. It also avoid the effects of steroid therapy and immunosuppressive drugs.

## Abbreviations

AIHA: Autoimmune hemolytic anemia
DAT: Direct antiglobulin test
Hb: Hemoglobin
Ht: Hematocrit
IVIG: Intravenously administered immunoglobulin
RBCs: Red blood cells
